# Poster Session I - A120 PRELIMINARY RESULTS ON THE FEASIBILITY AND ACCEPTABILITY OF A MENTAL HEALTH PATHWAY FOR IBD

**DOI:** 10.1093/jcag/gwaf042.120

**Published:** 2026-02-13

**Authors:** B Elchitz, E Rahime, R Kaur, S Raiesdana, K D Chappell, Y Zhang, F Peerani, K Kroeker, D Kao, M Gozdzik, F Hoentjen, B Halloran, K Wong, C Seow

**Affiliations:** Faculty of Kinesiology, University of Calgary, Calgary, AB, Canada; University of Alberta Department of Medicine, Edmonton, AB, Canada; University of Alberta Department of Medicine, Edmonton, AB, Canada; University of Alberta Department of Medicine, Edmonton, AB, Canada; University of Alberta Department of Medicine, Edmonton, AB, Canada; University of Alberta Department of Medicine, Edmonton, AB, Canada; University of Alberta Department of Medicine, Edmonton, AB, Canada; University of Alberta Department of Medicine, Edmonton, AB, Canada; University of Alberta Department of Medicine, Edmonton, AB, Canada; University of Alberta Department of Medicine, Edmonton, AB, Canada; University of Alberta Department of Medicine, Edmonton, AB, Canada; University of Alberta Department of Medicine, Edmonton, AB, Canada; University of Alberta Department of Medicine, Edmonton, AB, Canada; Faculty of Kinesiology, University of Calgary, Calgary, AB, Canada

## Abstract

**Background:**

People living with Inflammatory Bowel Disease (IBD) face a disproportionate burden of mental health concerns, yet access to timely and effective psychological support remains limited and costly. Mental health pathways have the potential to improve accessibility, but their feasibility and acceptability in this population remain unclear.

**Aims:**

This prospective, single arm intervention study evaluated the feasibility of a mental health pathway utilising digital tools among individuals with IBD.

**Methods:**

Adults with IBD were recruited from two academic IBD clinics between May and August 2025, and enrolled into a multi-timepoint study involving digital mental health tools, self-reporting diaries, with follow-up validated questionnaires (PHQ-9, GAD-7, PSSQ, FACIT-F, SIBDQ, WHO-5, WPAI, Social Connections) at baseline, one, two, and three months. Feasibility was assessed through recruitment, retention, and withdrawal data. Acceptability was evaluated using the Theoretical Framework Acceptability (TFA) questionnaire.

**Results:**

Of 179 eligible individuals, 116 participants completed screening, with 55 active participants. A total of 62 participants withdrew, 48 (77%) after the screening visit, 12 (20%) after one month, and 2 (3%) after two months; with 30 (48%) completing the withdrawal survey. Among withdrawals, 37% reported the study was too time consuming, 16% cited changes in personal circumstances, the remainder noted misunderstanding of the study, preference for in-person therapy, email burden, or added stress related to digital activities. Univariate analysis showed the median age of active participants were younger (32 years, range: 18-74) compared to those who withdrew (37 years, range: 18–70; p = 0.02). Each additional year of age was associated with 3% increased risk of withdrawal (OR 1.03; 95% CI: 1.00–1.05; p = 0.03). Participant sex and other demographic and clinical factors including disease duration, baseline GAD-7, PHQ-9 and SIBDQ were not significantly associated with study withdrawal. Regarding acceptability, most found the pathway helpful, fair, and confidence-building for self-management, with minimal interference in other priorities.

**Conclusions:**

Preliminary findings support the feasibility and acceptability of a digital mental health pathway among active participants with IBD. Study withdrawal was associated with increasing age but retention and acceptance maintained in younger individuals. Strategies to reduce burden and enhance clarity of study expectations may improve retention.

**Funding Agencies:**

Alberta Innovates

